# Adjuvant Therapy for Thymic Carcinoma – A Decade of Experience in a Taiwan National Teaching Hospital

**DOI:** 10.1371/journal.pone.0146609

**Published:** 2016-01-12

**Authors:** Yen-Han Tseng, Yi-Hsuan Lin, Yen-Chiang Tseng, Yu-Chin Lee, Yu-Chung Wu, Wen-Hu Hsu, Sang-Hue Yen, Jacqueline Whang-Peng, Yuh-Min Chen

**Affiliations:** 1 Department of Chest Medicine, Taipei Veterans General Hospital, Taipei, Taiwan, Republic of China; 2 School of Medicine, National Yang-Ming University, Taipei, Taiwan, Republic of China; 3 Department of Family Medicine, Taipei Hospital, Ministry of Health and Welfare, New Taipei, Taiwan, Republic of China; 4 Department of Surgery, Kaohsiung Veterans General Hospital, Pingtung Branch, Pingtung, Taiwan, Republic of China; 5 Sijhih Cathay General Hospital, New Taipei, Taiwan, Republic of China; 6 Division of Thoracic Surgery, Department of Surgery, Taipei Veterans General Hospital, Taipei, Taiwan, Republic of China; 7 Department of Oncology, Taipei Veterans General Hospital, Taipei, Taiwan, Republic of China; 8 Taipei Cancer Center, Taipei Medical University, Taipei, Taiwan, Republic of China; Faculté de médecine de Nantes, FRANCE

## Abstract

**Background:**

Thymic carcinomas are rare tumors for which surgical resection is the first treatment of choice. The role of adjuvant treatment after surgery is unknown because of limited available data. The present study evaluated the efficacy of post-surgery adjuvant chemotherapy or radiotherapy in patients with thymic carcinoma.

**Methods:**

To evaluate the role of adjuvant therapy in patients with thymic carcinoma, we retrospectively reviewed the records of patients with thymic carcinoma who were diagnosed and treated between 2004 and 2014.

**Results:**

Among 78 patients with thymic carcinoma, 30 patients received surgical resection. Progression-free survival (PFS) and overall survival (OS) were significantly longer among these patients than among patients who received other treatments (PFS: 88.4 months *vs* 9.1 months, *p*<0.001; OS: 134.9 months *vs* 60.9 months; *p* = 0.003). Patients with stage III thymic carcinoma who received surgery had a longer OS than patients who did not receive surgery (70.1 months *vs* 23.9 months; *p* = 0.017, n = 11). Among 47 patients with stage IV carcinoma, 12 patients who received an extended thymothymectomy had a longer PFS than 35 patients who did not receive surgery (18.9 months *vs* 8.7 months; *p* = 0.029). Among 30 patients (with stage I- IV carcinoma) who received primary lesion surgery, 19 patients received an R0 resection and 9 patients of the 19 patients received adjuvant radiotherapy. These patients had longer PFS (50.3 months) than 2 patients who received adjuvant chemotherapy (5.9 months) or 4 patients who received concurrent chemoradiotherapy (7.5 months) after surgery (*p* = 0.003).

**Conclusions:**

Surgical resection should be considered for patients with thymic carcinoma, even for patients with locally advanced or stage IV carcinoma. Adjuvant radiotherapy resulted in a better PFS after R0 resection.

## Introduction

Thymic carcinomas are rare neoplasms with a poor prognosis and a 5-year survival rate of approximately 30%-60%.[1, 2] Because of the rarity of this disease, data for treatment are limited.[2, 3] For resectable thymic carcinomas, surgical intervention is usually the first line of treatment.[4] On the other hand, radiotherapy and/or chemotherapy are considered first-line treatments when the tumor is locally advanced or metastatic.[5]

Previous studies documented that surgical resectability was an important prognostic factor. The patients who underwent total resection had a better prognosis than patients who underwent subtotal resection and patients who were inoperable.[6] In additionally, an international study (n = 1042) demonstrated that R0, R1, and R2 resections result in significantly different 5-year OS rates of 70%, 55%, and 48%, respectively.[1] The study also reported that adjuvant therapy with radiotherapy instead of chemotherapy may prolong patient survival. However, the optimal adjuvant therapy after surgical resection is not well established and remains controversial.

The aim of this study was to compare the outcomes of different treatment modalities- surgery, chemotherapy, radiotherapy, and concurrent chemoradiotherapy (CCRT) in patients with surgically resectable and unresectable thymic carcinoma.

## Materials and Methods

### Study design and patients

We retrospectively reviewed and analyzed the records and image files of patients with thymic carcinoma who were diagnosed and treated at Taipei Veterans General Hospital (Taipei, Taiwan) between 2004 and 2014. Pathologists confirmed all diagnoses. The clinical staging system at the time of diagnosis was based on the National Comprehensive Cancer Network guidelines.[7] We compared patients who received different treatment modalities, which included surgery, chemotherapy, radiation therapy, or CCRT. This review was approved by the institutional review board of Taipei Veterans General Hospital (No.: 2015-02-008AC). All patient records were de-identified before analysis, and therefore, informed consent was not required.

Clinical characteristics, such as each patient’s age, sex, Eastern Cooperative Oncology Group (ECOG) performance status, and smoking history were recorded. Patients were classified as a “never-smoker” (i.e. the patient had smoked less than 100 cigarettes in his or her lifetime) or as a “smoker”.

### Efficacy evaluation

A chest computed tomography scan was performed within 3 weeks before the patient started chemotherapy, and then every 2 to 3 months, or performed when confirmation of treatment response or disease progression was required. Tumor response was assessed using the Response Evaluation Criteria in Solid Tumors (RECIST, version 1.1).[8] Progression-free survival (PFS) was calculated from the date of surgery or the administration of the first dose of chemotherapy or radiotherapy until the earliest sign of disease progression, as determined by RECIST, or until death from any cause. If disease progression had not occurred by the time of the last follow-up visit, the PFS was censored at that time. Survival was measured from the date of surgery or administration of the first dose of chemotherapy or radiotherapy until the date of death or the last follow-up visit. Overall survival (OS) was censored for patients who remained alive at the time of the last follow-up visit.

### Statistical analysis

All categorical variables were analyzed with Chi-square tests or the Fisher exact test. Mann Whitney U tests were conducted for continuous variables when comparing two groups. The chemotherapy response rate was compared between treatment groups. The median PFS and OS were calculated using the Kaplan Meier method and compared using the log-rank test. All analyses of the differences were 2-tailed, and *p* < 0.05 was considered statistically significant. The statistical analyses were performed using SPSS software (version 19.0; SPSS Inc., Chicago, IL).

## Results

### Patients

Thymic carcinomas were diagnosed and treated in 78 patients between 2004 and 2014. At the initial diagnosis, 3 patients had stage I disease, 13 had stage II disease, 15 had stage III disease, and 47 had stage IV disease. All patients received treatment, including 30 patients who received surgery at the beginning of treatment.

### Patients who received surgical treatment versus non-surgical treatment

Among 30 patients who received primary lesion surgery, 19 patients received an R0 resection, 6 patients received an R1 resection, and 5 patients received an R2 resection (Table 1). Of the patients who received R0 resections, patients who received radiation therapy alone had a longer PFS than patients who received chemotherapy or CCRT (*p* = 0.003) (Table 2). Of those patients who received R1 resections, 2 patients received CCRT and 4 received radiation therapy alone. Only one patient in the radiation group died after follow up for 134.9 months. The remaining 3 patients are still alive at their last follow up. The follow-up time was 11.4 months and 51.3 months, for 2 patients in the CCRT group, respectively, with neither patient exhibiting signs of disease progression.

**Table 1:** The TNM staging for patients with thymic carcinoma (n = 78).

TNM staging	R0 resection* (n = 19)	R1 resection* (n = 6)	R2 resection* (n = 5)	No surgery (n = 48)
**1, n = 3**	2	0	0	1
**2, n = 13**	10	1	1	1
**3, n = 15**	3	1	0	11
**4, n = 47**	4	4	4	35

*Primary lesion

**Table 2:** Postoperative treatment modalities administered after R0 resections.

R0 resection	CCRT (n = 4)	C/T (n = 2)	R/T (n = 9)	No treatment (n = 3)	*p* value
**Sex (M)**	3 (75%)	0 (0%)	4 (44.4%)	2 (66.7%)	0.328
**Smoking**	0 (0%)	0 (0%)	3 (30%)	0 (0%)	0.031
**ECOG**					0.442
** 0**	1 (25%)	0 (0%)	1 (25%)	0 (0%)	
** 1**	3 (75%)	0 (0%)	2 (50%)	1 (33.3%)	
** 2**	0 (0%)	1 (50%)	1 (25%)	0 (0%)	
**Staging**					0.190
** 1**	0 (0%)	0 (0%)	1 (11.1%)	1 (33.3%)	
** 2**	1 (25%)	0 (0%)	7 (77.8%)	1 (33.3%)	
** 3**	2 (50%)	1 (50%)	0 (0%)	0 (0%)	
** 4**	1 (25%)	1 (50%)	1 (11.1%)	1 (33.3%)	
**PFS**	7.5 (5.1–9.8)	5.9 (5.9)	50.3 (18.9–88.4)	Not yet	0.003

The data are presented as the number (percentage) or as the median (range)

CCRT = concurrent chemoradiotherapy; ECOG = Eastern Cooperative Oncology Group; C/T = chemotherapy; PFS = progression free survival; R/T = radiotherapy.

Among the patients who received R2 resection, 3 patients received CCRT and 2 patients received chemotherapy. The median PFS was 8 months in the CCRT group and 15.4 months in the chemotherapy group.

The median PFS and OS were longer and the 2-year and 5-year survival rates were higher in patients who received an R0 resection than in patients who did not receive surgery. Among patients who received an R1 resection, the PFS was significantly longer than in patients who did not receive surgery; however, no significant differences were detected between these groups for OS or for the 2-year and 5-year survival rates. There were also no significant differences in the PFS or OS or in the 2-year and 5-year survival rates between patients who received an R2 resection and patients who did not receive surgical treatment (Table 3).

**Table 3:** The survival status of patients who received an R0, R1, or R2 resection versus patients who received no surgical treatment.

			*p* value
**R0 *vs* no surgery**	R0 resection (n = 19)	No surgery(n = 48)	
** Median PFS (months)**	88.4	9.1	0.001
** 95% CI**	24.3–152.6	8.5–9.7	
** Median OS (months)**	Not yet	60.9	0.001
** 95% CI**		18.2–103.6	
** 2-year survival**	11 (57.9%)	19 (39.6%)	0.040
** 5-year survival**	8(42.1%)	7(14.6%)	0.004
**R1 *vs* no surgery**	R1 resection (n = 6)	No surgery (n = 48)	
** Median PFS (months)**	Not yet	9.1	0.005
** 95% CI**		8.5–9.7	
** Median OS (months)**	134.9	60.9	0.142
** 95% CI**	134.9	18.2–103.6	
** 2-year survival**	3 (50.0%)	19 (39.6%)	0.335
** 5-year survival**	2 (33.3%)	7 (14.6%)	0.189
**R2 *vs* no surgery**	R2 resection (n = 5)	No surgery (n = 48)	
** Median PFS (months)**	8.0	9.1	1.000
** 95% CI**	0–19.7	8.5–9.7	
** Median OS (months)**	Not reached	60.9	0.990
** 95% CI**		18.2–103.6	
** 2-year survival**	3 (60.0%)	19 (39.6%)	0.303
** 5-year survival**	1 (20.0%)	7 (14.6%)	0.871

The data are presented as the number (percentage), unless otherwise indicated

CI = confidence interval; OS = overall survival; PFS = progression free survival.

### Stage I thymic carcinoma

Among 3 patients with stage I carcinoma, 2 (66.7%) patients received an R0 resection. One patient received adjuvant radiotherapy after surgery and had a PFS of 88.43 months. The second patient did not receive adjuvant therapy and the disease did not progress until their 1-month follow-up. The remaining 1 patient, who died 2.1 months after diagnosis, received only radiotherapy because of a poor general condition.

### Stage II thymic carcinoma

Among 13 patients with stage II carcinoma, 12 patients received surgery (Table 4). Ten surgeries were R0 resections, 1 surgery was an R1 resection, and 1 surgery was an R2 resection. Seven patients of 10 patients who received R0 resections also underwent adjuvant radiotherapy alone. One patient of the 10 patients who received an R0 resection subsequently exhibited disease progression after 43.4 months, whereas the remaining 9 patients did not show progression after a median follow up of 63.1 months (range, 1.6–76.7 months). The patient who received the R1 resection and adjuvant radiotherapy alone achieved a PFS and OS of 134.9 months. The patient who received the R2 resection and salvage CCRT achieved a PFS of 28.3 months. The patient was still alive after follow up for 31.8 months.

**Table 4:** Survival status by staging and surgery.

	Surgery	No surgery	*p* value
**All**	*N* = 30	*N* = 48	
** PFS (months)**	88.4	9.1	0.000
** 95% CI**	9.9–166.9	8.5–9.7	
** OS (months)**	134.9	60.9	0.003
** 95% CI**		18.2–103.6	
** 2-year survival**	17 (56.7%)	19 (39.6%)	0.073
** 5-year survival**	11 (36.7%)	7 (14.6%)	0.008
**Stage I**	N = 2	N = 1	
**Stage II**	N = 12	N = 1	
** PFS (months)**	62.4	38.9	0.069
** 95% CI**	33.6–79.9	38.9	
** OS (months)**	Not long enough	Not long enough	
** 95% CI**			
** 2-year survival**	9 (75.0%)	1 (100%)	1.000
** 5-year survival**	7 (58.3%)	0 (0%)	0.462
**Stage III**	N = 4	N = 11	
** PFS (months)**	5.9	16.6	0.992
** 95% CI**	4.7–7.2	6.3–26.9	
** OS (months)**	70.1	23.9	0.017
** 95% CI**	26.3–120.9	10.7–45.2	
** 2-year survival**	2 (50.0%)	5 (45.5%)	0.645
** 5-year survival**	2 (50.0%)	2 (18.2%)	0.275
**Stage IV**	N = 12	N = 35	
** PFS (months)**	18.9	8.7	0.029
** 95% CI**	8.0–25.6	6.0–11.5	
** OS (months)**	12.1	13.5	0.428
** 95% CI**	10.3–33.6	17.3–40.0	
** 2-year survival**	5 (41.7%)	13 (37.1%)	0.712
** 5-year survival**	1 (8.3%)	5 (14.3%)	0.450

The data are presented as the number (percentage), unless otherwise indicated

CI = confidence interval; OS = overall survival; PFS = progression-free survival

### Stage III thymic carcinoma

Among 15 patients with stage III carcinoma, 4 (21.2%) patients received surgery (Table 4). Among these 4 patients, 3 patients received an R0 resection and 1 patient received an R1 resection. Among the 3 R0 patients, 2 patients received adjuvant CCRT and 1 patient received adjuvant chemotherapy. The PFS was worse in patients who received surgery than in patients who did not receive surgery, although the difference was not significant (median time, 5.9 months vs. 16.6 months, respectively; *p* = 0.992). By contrast, the OS was longer in patients who received surgery than in patients who did not receive surgery (median, 70.1 months vs. 23.9 months, respectively; *p* = 0.017). No difference in the 2-year or 5-year survival rates was detected between patients who received surgery and patients who did not. The median PFS was 5.5 months (range, 5.1–5.9) among patients who received an R0 resection. All 3 patients who had an R0 resection remained alive after a follow-up time of 2.9 months, 12.6 months, and 122.1 months, respectively. The patient who received an R1 resection had adjuvant radiotherapy, and the disease had not progressed after a follow up of 116.7 months.

### Stage IV thymic carcinoma

Among 47 patients with stage IV carcinoma patients, 12 (25.5%) patients received primary lesion surgery (Table 4). Four patients received a primary lesion R0 resection, 4 patients received a primary lesion R1 resection, and 4 patients received a primary lesion R2 resection. The overall PFS was significantly longer in patients who received surgery than in patients who did not receive surgery (18.9 months *vs* 8.7 months, respectively; *p* = 0.029). By contrast, no significant differences in OS or in the 2-year and 5-year survival rates were detected between patients who underwent surgery and patients who did not. The median PFS was 14.5 months for patients who received R0 resections and 5.3 months for patients who received R2 resection. All patients who received R1 resection did not progress after follow up for 3.3–51.3 months.

### Postoperative adjuvant treatment

Twenty-six patients received postoperative adjuvant treatment and 4 patients did not. Among the patients who received adjuvant treatment, 8 patients received CCRT, 5 received chemotherapy, and 13 patients received radiotherapy. The OS and the 2-year and 5-year survival rates were not statistically different between these subgroups of patients. However, the PFS was significantly longer among patients who received adjuvant radiotherapy alone (*P* = 0.018) (Table 5), compared to other treatments.

**Table 5:** Postoperative treatment after surgery.

	CCRT (n = 8)	C/T (n = 5)	R/T (n = 13)	No treatment (n = 3)	*p* value
**Age (y)**	54.6 (50.3–58.9)	47.6 (32.5–62.7)	58.3 (50.9–65.7)	68.3 (49–87.6)	0.064
**Sex (M)**	6 (75.0%)	2 (40.0%)	6 (46.2%)	2 (66.7%)	0.508
**Smoking**	2 (25.0%)	0 (0%)	3 (23.1%)	0 (0%)	0.234
**ECOG**					0.864
** 0**	1 (12.5%)	1 (33.3%)	1 (12.5%)	0 (0%)	
** 1**	6 (75.0%)	1 (33.3%)	6 (75.0%)	1 (100%)	
** 2**	1 (12.5%)	1 (33.3%)	1 (12.5%)	0 (0%)	
**Staging**					0.172
** 1**	0 (0%)	0 (0%)	1 (7.7%)	1 (33.3%)	
** 2**	2 (25.0%)	0 (0%)	8 (61.5%)	1 (33.3%)	
** 3**	2 (25.0%)	1 (20.0%)	1 (7.7%)	0 (0%)	
** 4**	4 (50.0%)	4 (80.0%)	3 (23.1%)	1 (33.3%)	
**PFS**	6.6	9.8	65.6	23.8	0.018
** 95% CI**	1.7–28.5	0.1–12.9	34.4–83.6	0–54.6	
**OS**	12.0	17.8	65.6	Not long enough	0.443
** 95% CI**	0.8–47.0	0–97.0	35.4–84.8		
**2-year survival**	3 (37.5%)	2 (40.0%)	10 (76.9%)	1 (33.3%)	0.373
**5-year survival**	1 (12.5%)	1 (20.0%)	8 (61.5%)	0 (0%)	0.121

The data are presented as the number (percentage), unless otherwise indicated. The total number of patients is 29 patients.

CCRT = concurrent chemoradiotherapy; CI = confidence interval; C/T = chemotherapy; ECOG = Eastern Cooperative Oncology Group; R/T = radiotherapy.

### Efficacy of chemotherapy in patients with measurable lesions

Fifty-five patients received chemotherapy, which included 53 patients who received platinum-based chemotherapy. After excluding an adjuvant setting or concurrent chemoradiotherapy, the objective response rate was 23.1% for 13 patients who had measurable lesions. The median PFS was 8.7 months for 13 patients treated with platinum-based chemotherapy alone, and the OS was 13.2 months. Six patients received etoposide plus cisplatin treatment and 3 (50%) patients had a partial response. Five patients received cyclophosphamide, doxorubicin (Adriamycin), and cisplatin treatment. No objective response occurred.

## Discussion

Our study demonstrated that the OS of patients with stage III thymic carcinoma was longer in patients who received surgery than in patients who did not receive surgery. Furthermore, the PFS of patients with stage IV thymic carcinoma was longer when patients received primary lesions resections. However, patients who received primary lesion R1 or R2 resections did not exhibit a significantly longer PFS or OS or a higher 5-year survival rate than patients who did not receive surgical resection.

Weksler et al. suggest that surgery is the preferred treatment for thymic carcinoma and that complete resection imparts the best survival.[2] Venuta et al. showed that complete resection is the key factor for the cure of thymic tumors and should be considered for any tumor stage.[9] Many other studies also suggest that surgery is the cornerstone of therapy for thymic tumors.[3, 6, 10, 11] In support of these studies, our data demonstrated that surgery resulted in better survival in patients with thymic carcinoma. In the current study, patients with stage III and IV thymic carcinoma exhibited better survival when they received surgical resection of the primary lesion. However, our data did not detect a significant survival advantage of surgery for patients with stage I and stage II carcinoma. The finding is probably because of the limited patient number and the short follow-up time.

Tumor resectability is also one of the most important prognostic factors for patients with thymic carcinoma.[12] Ahmad et al. demonstrated that patients who received R0, R1, and R2 resections had significantly different survival time; however, limited data were presented for patients who did not receive surgical resection.[1] In the current study, patients who received an R0 resection had significantly longer PFS and OS as long as higher 5-year survival rates than patients who did not receive surgery. By contrast, for all tumor stages, patients who received an R2 resection did not exhibit significantly better survival than patients who did not receive surgical intervention. The PFS was longer among patients who received an R1 resection than those among patients who did not receive surgery. However, the OS and the 2-year and 5-year survival times were not significantly different between patients who received an R1 resection and patients who did not receive surgery. These results have never been reported previously.

The use of adjuvant treatment for thymic carcinoma is controversial. Sakai et al. suggest that surgical resection without adjuvant chemotherapy or radiotherapy could be considered for the early stages of thymic carcinoma.[13] No local recurrence or metastasis was noted in their study.[13] Song et al. suggest that radiation therapy tends to decrease local recurrence or distant recurrence rates in patients with all tumor stages.[4] In additionally, Ahmad et al. demonstrated that chemotherapy and radiation therapy were both associated with a better OS in patients with thymic carcinoma.[1] Our data demonstrated that patients who underwent R0 resection and received adjuvant radiotherapy had longer PFS than patients who received adjuvant chemotherapy or CCRT alone.

The optimal regimen of chemotherapy for thymic carcinomas remains controversial. Current National Comprehensive Cancer Network guidelines suggest several combination regimens; such as cisplatin plus doxorubicin and cyclophosphamide (CAP), cisplatin plus doxorubicin, cyclophosphamide, and vincristine (ADOC), etoposide plus cisplatin (EP), etoposide plus ifosfamide and cisplatin (VIP), and carboplatin plus paclitaxel.[14] Most published studies enrolled small numbers of patients, thereby making it difficult to properly assess the benefits of these regimens.[15–17] Okuma et al. performed a systemic review of published clinical trials and found that platinum with anthracycline-based chemotherapy was the best combination regimen for treating thymomas and that cisplatin-based chemotherapy was superior to a carboplatin-based regimen for treating advanced thymic carcinoma.[18] There was no significant difference in the PFS or OS among our patients who received first-line chemotherapy with CAP, EP, or platinum-based regimens and patients who received non-CAP, non-EP, or non-platinum-based regimens, respectively.

This study had several limitations. First, it was a retrospective study with a small patient numbers. Therefore, selection bias undoubtedly exists. In the future, a prospective study with a large patient cohort should be performed. Second, our longest follow-up time was 153 months. A longer follow-up time for all patients is needed to fully assess the PFS and OS associated with the treatment of thymic carcinoma.

## Conclusions

Surgical resection should be considered for patients with advanced or stage IV thymic carcinomas. Patients receiving adjuvant radiotherapy exhibited a better PFS after R0 resection; therefore, this surgery should be a routine procedure or a prospective adjuvant radiotherapy trial should be conducted.

## Supporting Information

S1 Data This is the minimal data set. (XLSX)
